# Coenzyme Q10 Modulates Remodeling Possibly by Decreasing Angiotensin-Converting Enzyme in Patients with Acute Coronary Syndrome

**DOI:** 10.3390/antiox7080099

**Published:** 2018-07-25

**Authors:** Ram B. Singh, Jan Fedacko, Viliam Mojto, Dominik Pella

**Affiliations:** 1 Halbergt Hospital and Research Institute, Moradabad 244001, India; 2 Department of Internal Medicine, PJ Safaric University, 1040 11 Kosice, Slovakia; janfedacko@hotmail.com; 3 Third Interna Clinik, Faculty of Medicine, Comenius University, 833 05 Bratislava, Slovakia; viliam.mojto@kr.unb.sk; 4 Department of Cardiology, East Slovak Institute of Cardiovascular Sciences, 1040 11 Kosice, Slovakia; dominik.pella@gmail.com

**Keywords:** remodeling, bioenergetics, heart failure, antioxidants, angiotensin-converting enzyme, echocardiography

## Abstract

The study aims to examine the effects of coenzyme Q10, (a bioenergetic antioxidant), on the indexes of left ventricular remodeling, oxidative damage, and angiotensin-converting enzyme (ACE) level after acute myocardial infarction (AMI) with left ventricular dysfunction. In a double blind, randomized, placebo-controlled, parallel group study (a retrospective analysis of an earlier trial) in 55 patients with left ventricular ejection fraction <50% after AMI, the effects of coenzyme Q10 (120 mg/day) or placebo were studied for 24 weeks. Two-dimensional echocardiography was performed at discharge, (approximately 5–10 days after admission) and at 6 months after AMI. The results revealed that wall thickness opposite the site of infarction decreased from (mean ± standard deviation (SD)) 12.2 ± 2.0 mm to 10.0 ± 1.8 mm with coenzyme Q10 compared with 12.8 ± 2.2 mm to 13.3 ± 2.3 mm with placebo (*p* < 0.01). Left ventricular mass changed from 236 ± 72 g to 213 ± 61 g with coenzyme Q10 compared with 230 ± 77 g to 255 ± 86 g with placebo (*p* < 0.01). Treatment with coenzyme Q10 also prevented alteration of sphericity index which is a ratio of the long and short axis of the left ventricle (which changed from 1.61 ± 0.28 to 1.63 ± 0.30 with coenzyme Q10 compared with 1.61 ± 0.32 to 1.41 ± 0.31 with placebo (*p* < 0.05)). Coenzyme Q10 also prevented alteration of the wall thickening abnormality at the infarct site, which changed from 9.4 ± 3.0 cm^2^ to 9.1 ± 2.8 cm^2^ compared with 10.1 ± 3.1 to 13.7 ± 4.2 cm^2^ with placebo (*p* < 0.05). End diastolic and systolic volumes also showed significant reduction with coenzyme Q10 compared to placebo. The serum level of ACE showed significant decline in the coenzyme Q10 group compared to the control group. Treatment with coenzyme Q10 early after AMI causes attenuation of left ventricular remodeling and decreases the serum ACE level in patients with left ventricular dysfunction.

## 1. Introduction

A majority of the complications of acute myocardial infarction (AMI) are due to the presence of left ventricular dysfunction which appear to be on account of reperfusion-induced free radical damage, lipid peroxidation and decreased energy production as well as due to antioxidant vitamin and coenzyme Q10 deficiency [1,2,3,4]. The mechanisms of left ventricular dysfunction may be related to loss of functioning myocardium from necrosis and by the adverse effects of pathologic remodeling [2,3]. Cardiac remodeling may be defined as a group of molecular, cellular and interstitial alterations that manifest clinically as changes in size, mass, geometry and function of the heart after injury [1]. These changes in ventricular remodeling in conjunction with antioxidant vitamin and coenzyme Q10 deficiency, may result in poor prognosis because of its association with ventricular dysfunction [1,2,3,4].

An expansion of infarct size, hypertrophy of non-infarcted myocardium, increased total left ventricular mass and alteration of left ventricular geometry are common in patients with left ventricular remodeling [5,6,7,8,9,10]. These structural changes in the myocardium are associated with progressive left ventricular dysfunction and heart failure which is the major cause of morbidity and mortality in patients with AMI. Therefore, any agent which can prevent remodeling in patients with AMI would be an important therapeutic aid, for prevention of complications alter AMI [6,7,11,12]. Singal and his group have demonstrated that oxidative stress may be important in the pathogenesis of remodeling which may begin via sub-cellular remodeling leading to heart failure [8,12,13]. Treatment with coenzyme Q10 in heart failure prevents myocardial cell damage and repairs coenzyme Q10 deficiency in both blood and myocardial tissue resulting into significant improvement in heart failure [14,15,16,17,18,19,20]. The degree of coenzyme Q10 deficiency has been found to correlate directly with the degree of impairment in left ventricular function [20]. Coenzyme Q10 is also regenerable, bioenergetic, a powerful antioxidant and an ATP sparing agent capable of protecting cell structures from oxidative damage during ischemia and reperfusion injury [21,22]. There is evidence that CoQ10 may prevent the development of cardiac remodeling, and fibrosis in isoprenaline-administered rats, which generates the possibility that it might prevent heart failure [2]. Our study is unique, because no study has examined the role of coenzyme Q10 in the prevention of remodeling in patients with AMI. We have reported that hydrosoluble and rapidly absorbed coenzyme Q10 administration early after AMI and thrombolysis was safe, and reduced cardiac events within 4 weeks of follow up [5]. In this retrospective study, we have examined the possible role of coenzyme Q10 on indexes of remodeling after AMI in those with impaired left ventricular function.

## 2. Subjects and Methods

The subjects for the present study were a subgroup from the larger data base of our previous study [5]. It was a double blind, randomized, parallel group, placebo-controlled trial. The subjects were stratified for location of infarction (anterior or posterior) and complications (hypotension, arrhythmia and heart failure) and randomized by selecting cards marked group A and B, kept in sealed envelops. Subjects admitted to the cardiac care unit with classical manifestations qualified for the diagnosis of AMI such as chest pain (>30 min duration) electrocardiographic changes and serum concentration of creatine kinase and MB isoform (double the rise of normal) consistent with the diagnosis. The electrocardiographic changes were symmetric ST segment elevation of 1 mm from baseline in limb leads or of 2 mm in chest leads or T wave inversions with or without Q waves. The study was approved by the ethics committee of our center and all the subjects gave informed consent in writing. Exclusion criteria were blood urea nitrogen >20 mg/dL, malignancy, cardiogenic shock as described elsewhere [5]. Patients developing re-infarction during the follow up of 6 months were recorded.

All the patients presented within 72 h of the onset of symptoms of AMI. All the patients were advised aspirin and the decision for heparin, thrombolysis, beta blockers, angiotensin-converting enzyme (ACE) inhibiters was left entirely to the judgement of the admitting physician. Subsequently after randomization, subjects were administered either two capsules of coenzyme Q10 (30 mg each capsule) twice daily (120 mg of coenzyme Q10/day) to group A or two capsules twice daily of B vitamins to control group B (each capsule containing thiamine mononitrate IP 3 mg, riboflavine IP 3 mg, pyridoxine hydrochloride 1 mg and nicotinamide IP 25 mg). The control group was administered B vitamins, because the ethics committee did not allow us to give inactive placebo. B vitamins are known to provide some marginal benefits in decreasing homocysteines that are high and may increase the risk in AMI patients. The active and control capsules were not truly identical, and therefore both groups of patients were instructed not to show and compare their capsules with those of other patients, not to show them to their doctor, and both groups met separately. Both groups were told that they were being given vitamin capsules for the treatment of AMI to maintain the blind nature of the trial. The test capsules were provided to patients by the pharmacist in sealed containers that were identical in all respects. Compliance was monitored by counting the number of capsules returned by the patients on follow up visits during the period of 6 months. End points were defined as cardiac events such as cardiac death, re-infarction, unstable angina and heart failure. Intake of furosemide, ACE inhibitors, digitalis, vasodilator were noted in both the groups. However, these end points were noted in both groups without discontinuation of treatment.

### 2.1. Two-Dimensional Echocardiography

The cardiologist performing two-dimensional M mode echocardiography before discharge and at 6 months after AMI with a digitized system was blind to treatment groups A and B. Multiple views were obtained from all the axes. Left ventricular wall thickness was measured in the short-axis view at the level of papillary muscles at end diastole. Left ventricular mass was calculated by the method used in previous studies [23]. The sphericity index was calculated as the rate of left ventricular diameters at end diastole in an apical 4-chamber view which is the vertical distance between the apical endo-cardium and mitral annulus and a short-axis view at the level of papillary muscle which is vertical distance between the septal and posterior endocardium [11]. Three cycles were measured for each assessment and an average was calculated of these values. The wall-thickening area abnormalities at the site of the AMI which corresponded with the site where acute electrocardiographic abnormalities appeared were manually traced by the electronic marker in the short-axis views at the level of papillary muscle. The endocardium was identified anterior or posterior to the papillary muscles depending on whether the infarction was anterior or inferior, respectively. The area of wall-thickening abnormality at the infarct site was then automatically calculated with the help of software (Ultramark 9, HDI, ATL, Berlin, Germany). Ectopic and post-ectopic beats were avoided. Qualitative assessment of left ventricular systolic function was made by the eye ball technique with two-dimentional images [24,25]. Quantitative assessment was made by measurement of ejection fraction and fractional shortening by using M-mode echocardiography. Ejection fraction was also measured using the Simposons byplane method [26]. For diastolic function, the E:A ratio was measured as described earlier [27,28].

### 2.2. Laboratory Data

A blood sample after at least 10 h of fasting was drawn at entry and then at 4 weeks, 12 weeks and 24 weeks of follow up in the morning and analyzed for blood count, hemoglobin, urea, glucose, vitamin, E and C, beta-carotine thiobarbituric acid reactive substances (TBARS), melondialdehyde and diene conjugates and angiotensin-converting enzyme [29,30,31,32,33,34]. The technician was blind to groups. All the vitamins and parameters of oxidative stress were assayed by colorimetric methods as given in detail in a previous publication [5]. The purpose was to find out the extent of oxidative damage and antioxidant status which may be altered due to AMI. Previous studies have shown that there is marked deficiency of vitamin A, E and C in patients with acute myocardial infarction(AMI), which may be due to oxidative stress caused by catecholamines and cortisol released during infarction [3,4,5]. It is possible that these antioxidant vitamins are consumed by the cardiomyocytes in an attempt to inhibit the free radical stress, resulting in their deficiency in patients with AMI.

### 2.3. Statistical Analysis

All the subjects were stratified at entry, hence data were normally distributed and homogeneous in both the groups. All discrete variables are expressed as numbers and percentages and continuous variables as mean ± standard deviation (SD). Only two tailed t test and a *p* value < 0.05 were considered significant. Analysis of variance followed by Tukey’s post-hoc test were performed to find out differences between two groups and level of significance.

## 3. Results

Of the 174 patients screened, 144 were randomized to intervention group A (*n* = 73) or placebo group B (*n* = 71). Mean age (mean ± SD) (48 ± 8.6 vs. 47.6 ± 8.2 years), body weight (65.0 ± 6 vs. 64.7 ± 6 kg), male sex (79.4 vs. 80.3%), and previous myocardial infarction (6.8 vs. 11.2%) showed no significant difference. AMI (78.0 vs. 78.8%), possible AMI (13.7 vs. 11.2%) and unstable angina (8.2 vs. 9.8%) were also comparable between the two groups. Anterior or universal infarction (64.4 vs. 66.2%), left ventricular enlargement (13.7 vs. 9.8%), elapsed time from symptom onset to intervention (42 ± 4.6 vs. 46 ± 4.8 h) and previous as well as drug therapy given at admission showed no significant differences. Mean heart rate (115 ± 18.6 vs. 118 ± 21.6 per min) and systolic (138 ± 20 vs. 140 ± 22 mmHg) and diastolic (88.6 ± 10.6 vs. 90.6 ± 12.3 mmHg) blood pressures were comparable between the two groups at randomization.

Of 144 patients, those on beta-blocker and ACE-inhibiters were excluded and 130 underwent echocardiography before discharge. Of these, 55 had left ventricular ejection fraction of <50% who were selected for further study and compared with the remaining 75 patients with left ventricular ejection fraction 50% and above (Table 1). These patients represented all of those sub-grouped patients with ejection fraction <50%. Table 1 and Table 2 reveal baseline characteristics of patients included and sub-grouped in the sub-study. Table 1 shows that there was a significantly greater (*p* < 0.02) frequency of previous coronary artery disease, anterior or universal infarction, ventricular enlargement at presentation, and larger infarct size based on creatine kinase levels in patients with left ventricular ejection fraction <50% compared to subjects with higher ejection fraction.

Table 2 shows that there were no significant differences between the intervention and control groups in patients with left ventricular ejection fraction <50% in regard to demographic data, particularly in relation to heart failure at presentation, site of infarction and infarct size. Antioxidant vitamin levels and oxidative damage at baseline showed no significant difference. ACE levels showed no significant difference in patients with left ventricular ejection fraction <50% in the intervention and control group (Table 2).

Only 10 patients (5 in each group) were administered streptokinase including 6 patients among those with left ventricular ejection fraction <50% due to delays in admission after chest pain. All patients were administered aspirin and 96% received either oral or intravenous nitrate. Oral or intravenous furosemide was administered in the acute phase to control left ventricular failure. Long-acting nitrates and ACE inhibitors were not received as the clinical requirement of these drugs was an end point and, moreover, the study was conducted in 1996–1997 when these agents were not commonly used in AMI. All patients of the intervention group were receiving 120 mg/day of coenzyme Q10 during the study.

The hemodynamic response was much better in the intervention group compared to the control group. Heart rate and systolic and diastolic blood pressure which were greater at entry to the study, showed significant decline in the coenzyme Q group compared to placebo group. The response was seen before discharge which persisted at 6 months (Table 3). Table 4 shows the end diastolic volume and end systolic volume in the intervention and control groups. In coenzyme Q10 treated patients, both end diastolic and end systolic volumes were significantly smaller at 6 months after AMI. compared to the control group. Coenzyme Q10 group showed a similar left ventricular wall thickness of the non-infarcted wall compared with patients treated with the placebo at pre-discharge baseline, but coenzyme Q10 produced a significant reduction of the non-infarcted wall thickness at 6 months compared with the placebo. Left ventricular mass also followed the same pattern on treatment with coenzyme Q10.

Coenzyme Q10 group showed a significant reduction in left ventricular mass which increased with the placebo (Table 4). Area of wall-thickening abnormality at the site of infarct showed significant reduction in the coenzyme Q10 group, whereas it increased in the placebo group between pre-discharge and 6 months. The sphericity index showed a significant decrease in the control group whereas coenzyme Q10 significantly attenuated this abnormality, favorably altering cardiac geometry without causing significant change in sphericity index. Ejection fractions that were lower initially in the two groups (41.6 + 5.2 vs. 42.6 + 5.6%) showed significant increase in the coenzyme Q10 group compared to the control group (54.5 + 8.5 vs. 38.5 + 4.6%, *p* < 0.05). There were slightly fewer cardiac deaths (4 vs. 9) and nonfatal infarction (6 vs. 11) in the intervention group compared to the control group, respectively.

Antioxidant vitamin E and C and beta-carotene showed a significant increase whereas thiobarbituric acid reactive substances, malon-dialdehyde and diene conjugates which are indicators of free radical damage, showed significant decline in the intervention group than control group. There was a significant decrease in the serum concentration of angiotensin-converting enzyme level in the coenzyme Q10 group compared to the control group (Table 5).

## 4. Discussion

This retrospective analysis shows, possibly for the first time in humans, that treatment with coenzyme Q10 in patients with ejection fraction <50% in the setting of AMI may be associated with significant reduction in left ventricular wall thickness and mass as well as in the area of wall-thickening abnormality at the site of the infarct compared to the control group. Treatment with coenzyme Q10 significantly attenuated the sphericity index favorably altering cardiac geometry. There was a significant decline in end diastolic and systolic volume in the intervention group compared to changes in the control group (Table 4). Echocardiography was performed by an external cardiologist who was kept blind to the groups and was not aware of the trial at the time of discharge of the patients from the hospital and 6 months after the AMI, to overcome the operator’s bias as well as inter- and intra-observer variability of echo parameters. Left ventricular end diastolic and systolic volumes and the extent and severity of wall-thickening abnormalities were different between coenzyme Q and placebo groups at discharge from the hospital, however no statistically significant differences were observed. Since these parameters were not obtained at the time of randomization, it is just possible that the placebo group was a higher risk group compared to treatment group resulting into more aggressive remodeling which is a limitation of our study. However, such a possibility is quite unlikely. The number of subjects were only 55 which is a weakness of our study; although, in demonstrating such original findings, similar numbers were also included in the carvidilol study (*n* = 45) [11]. Experimental studies using CoQ10 in AMI confirm our findings [2].

Secondly, the baseline characteristics at the time of randomization such as the presence of heart failure, thrombolysis, infarct size based on creatinine kinase and elapsed time from symptom onset to intervention showed no significant differences between the two groups. This study has shown significant benefits of coenzyme Q10 on markers of remodeling as well as on left ventricular dysfunction. These findings indicate that coenzyme Q10 had a substantial protective effect against progressive left ventricular remodeling after AMI. Remodeling associated with AMI is characterized by deleterious alterations of left ventricular size, shape and thickness involving the infarcted and non-infarcted segments of the myocardium [2,10]. Infarct size, infarct healing, left ventricular distending pressure, inotropic state, heart rate, heart rate variability and neuroendocrine activation are important factors in the pathogenesis of remodeling [8,9,10,11,12,13]. It is possible that these factors may have enhanced remodeling during acute ischemia, resulting in abnormal parameters of remodeling at baseline (Table 4). Prevention of left ventricular remodeling, therefore, is a major therapeutic goal after AMI because it delays the occurrence of heart failure. In patients with chronic heart failure, remodeling can be reversed but in AMI, progressive remodeling is only prevented, it is surprising, therefore, that the markers of remodeling actually improved compared to the baseline indicating that coenzyme Q10 might protect against pathogenetic factors of remodeling. Only some of the patients received ACE inhibitors and beta-blockers as the study was conducted earlier, which have been shown to be beneficial following AMI. The incremental benefit of ubiquinone over and above these agents would have produced the most interesting data. However three studies reported no benefit of ubiquinone on existing treatment of heart failure in subjects with low ejection fraction [35,36,37]. In one study atrial fibrillation was present and in two studies, the plasma levels of ubiquinone did not reach a desired level and both patients were suffering cardiomyopathy without AMI.

Kuklinski et al. [7] studied 61 patients with AMI randomized to receive either coenzyme Q10 (100 mg/day) with 100 mg of selenium or placebo for a period of one year. The treatment group showed no prolongation of the Q-T-interval whereas in the placebo group, 40% of subjects showed prolongation of the corrected Q-T-interval of greater than 440 milliseconds which is an indicator of heart rate variability and a risk factor of sudden cardiac death. There were more deaths in the intervention group compared to the control group after one year of follow up (6 vs. 1). Neither this study nor other studies examined the influence of coenzyme QI0 on systolic and diastolic dysfunction in the setting of AMI. However, several studies [14,15,16,17,18,19,20] have demonstrated the beneficial effects of coenzyme Q10 in chronic heart failure with predominant systolic dysfunction. Seven of these were double blind, placebo-controlled trials from 14 to 641 patients treated with coenzyme Q10 in doses from 60–200 mg/day for 1–12 months. In 5 of the studies, left ventricular ejection fraction has been evaluated either by systolic time intervals, impedence cardiography, randionuclide ventriculography or echocardiography. One study showed no benefit, in which a patient had atrial fibrillation [35]. In another study in which ejection fraction was very low, <25%, no benefit was noted with coenzyme Q10 or ACE-inhibitors [36]. The change in left ventricular function was large in the coenzyme Q10 group because treatment was given earliest during the development of the dysfunction which leads to chronic heart failure.

Munkholm et al. [14] administered coenzyme Q10 (200 mg/day) or placebo in 22 patients (including 16 patients of post-AMI) with heart failure for 12 weeks in a randomized, double-blind, controlled fashion. Left ventricular ejection fraction was <45% and left ventricular internal diameter in diastole was >60 mm. After 12 week, there was a significant decrease in mean pulmonary artery pressure at rest and mean capillary wedge pressure at work and a significant increase in stroke index at rest and work. The decrease in mean pulmonary artery pressure at work was insignificant, although no changes were noted in the placebo group. These findings indicate improvement in left ventricular function, although, no benefit was observed in ejection fraction in the coenzyme Q10 group compared to the control group.

Coenzyme Q10 is a potent antioxidant bio-energetic and ATP-sparing agent which is very rich in mitochondria of cardiac myocytes [14,15,16,17,18,19,20,21,22]. Treatment with coenzyme Q10 was associated with significant decrease in all the parameters of oxidative damage, ACE level and an increase in antioxidant vitamins as well as beneficial effects on heart rate and blood pressure (Table 3 and Table 5). The increase in antioxidant vitamins may occur as a recovery, from acute illness, due to their sparing in both the groups. The underlying mechanism of attenuation of remodeling by coenzyme Q10 in the setting of AMI appears to be multifactorial. It is possible that decreases in myocardial oxygen consumption, left ventricular load, neuro-hormonal activation, oxidative stress and hypertrophy and repair of coenzyme Q10 deficiency may combine to produce these beneficial effects of coenzyme Q10 [8,9,10,11,12,13,14,15,16,17,18,19]. There was a substantial benefit on left ventricular function and geometry, although the number of cases was small. Moreover, coenzyme Q10 was started on average after 42 h of the onset of chest pain and the echocardiogram was not performed until days 5–10, it may be implied that this magnitude of the effects of coenzyme Q10 is underestimated in our study.

Since increased activity of angiotensin II is the major factor responsible for heart failure, it is possible that coenzyme Q10 may benefit by decreasing ACE level and angiotensin II levels in patients with left ventricular dysfunction [38]. It is not clear how coenzyme Q10, which is normally rich in kidneys and adrenals, decreases ACE levels. It seems that repair of the deficit evoked by an acute event or increased availability of coenzyme Q10 may have modulated renin-angiotensin-aldosterone activity resulting in a decrease in ACE levels. It is possible, therefore, that in the presence of a full dose of ACE inhibitors, coenzyme Q10 may be less effective, since the greatest impact may be lost due to down regulation of the neuro-hormonal toxicity by the ACE inhibitors. However, the direct effect of coenzyme Q10 on mitochondrial function of kidneys, adrenals, brain and heart appears to be much greater than the indirect benefit due to reduction in ACE levels which is additive or synergestic. A single intravenous injection of solubilized coenzyme Q10 (30 mg/kg) at minute 60 effectively limited left ventricular damage and deterioration of function after coronary ligation in rats, which further confirms our results [39]. In a clinical study, patients with electrocardiographic ST segment elevation AMI, who had higher plasma coenzyme Q10 level, 1 month after primary angioplasty revealed better left ventricular (LV) performance at 6-month follow-up [40]. A higher plasma coenzyme Q10 level was associated with lower grade inflammatory and oxidative stress status indicating that plasma coenzyme Q10 level may serve as a novel prognostic biomarker of left ventricular systolic function after revascularization therapy for AMI.

Since coenzyme Q10 is the treatment of choice in patients with heart failure due to myocardial infarction [14,15,16,17,18], the possibility of early institution of coenzyme Q10 in the treatment of high risk patients may be of significant advantage. The results of our study and the role of coenzyme Q10 in heart failure [14,15,16,17,18] indicate that it is uniquely suitable for the management of high-risk patients of AMI. Treatment with coenzyme Q10 also repairs its deficiency which is common among these patients [14,15]. More studies in a large number of patients, in particular using 3D echocardiography, would be necessary to confirm our findings that coenzyme Q10 can prevent heart failure.

The dosage of coenzyme Q10 was 120 mg/day because it was a commonly administered dosage used in all previous studies showing clinical benefit in AMI and chronic heart failure [6,7]. There was no trial showing the use of higher doses of 200–600 mg/day of coenzyme Q10 in heart failure or AMI. Later studies revealed that the highest levels of coenzyme Q10 are observed when dosage is 100 mg taken in the morning and 100 mg in the evening [41]. Coenzyme Q10 is more effective if patients have a pre-existing deficiency of coenzyme Q10 in the tissues [42,43,44]. In a later study in patients with heart failure, higher dose of coenzyme Q10 (300 mg/day) showed significant decline on left ventricular function and cardiovascular events [44]. Heart failure has become a public health problem in every country of the world, hence further therapeutic advances are needed to prevent it [45,46].

## 5. Limitations

Due to a smaller number of cases, the study strength is optimal and it has limitations taking into account magnitude and sources of potential bias or imprecision in both directions. No attempt was undertaken to see whether CoQ10 interferes with the assay to determine ACE activity, due to our ignorance, which is a limitation of this study. Assays of plasma levels of CoQ10 and other B vitamins were not available in our laboratory, which is a limitation of this study. The control group was administered B vitamins, because the ethics committee did not allow us to give an inactive placebo, which is a limitation of this study. B vitamins are known to provide some marginal benefits in decreasing homocysteines.

## 6. Conclusions

In brief, the findings suggest that treatment with coenzyme Q10, administered early after AMI, may be protective against left ventricular remodeling in patients with persistent left ventricular dysfunction. The decline in ACE levels on treatment with coenzyme Q10 is a novel finding.

## Figures and Tables

**Table 1:** Baseline characteristics of patients with left ventricular ejection fraction <50% compared to subjects with ejection fraction 50% and above.

Characteristics	Ejection Fraction < 50%	Ejection Fraction 50% and above
*n*	55	75
Age (years)	48.5 ± 7.2	47.0 ± 6.4
Body weight (kg)	65.4 ± 5.5	64.3 ± 5.2
Body mass index (kgm^2^)	24.0 ± 1.4	23.2 ± 1.3
Men	45(81.8)n%	60(80.0)
Previous coronary artery disease	18(32.7) *	6(8.0)
Previous angina pectoris.	9(16.4)	4(5.3)
Known hypertension	26(47.2)	25(33.3)
Diabetes mellitus	18(32.7)	23(30.6)
Current smokers	18(32.7)	22(29.3)
Ex-smokers	6(10.9)	10(13.3)
Final diagnosis		
Acute myocardial infarction	48(87.2)	62(82.6)
Possible myocardial infarction	6(10.9)	10(13.3)
Unstable angina	1(1.8)	3(4.0)
Site of infarction		
Anterior or universal	50(90.9) *	45(60.0)
Inferior or posterior	5(9.1)	30(40.0)
Ventricular enlargement at presentation	27(49.2) *	18(24.0)
Thrombolysis	4(7.3)	8(10.6)
Elapsed time from symptom onset to the		
Treatment (hours)	40.1 ± 4.3	42.5 ± 4.6
Peak creatinine kinase (IU/L)	981 ± 185 **	628 ± 105

* *p* < 0.02, ** *p* < 0.01, *p* value was obtained by comparison of two groups. Values are mean ± standard deviation (SD) or number (%) in both groups.

**Table 2:** Baseline characteristics of patients with ejection fraction <50% in the intervention and control groups.

Clinical Data	Intervention Group (*n* = 27)	Control Group (*n* = 28)
Mean age	48.5 ± 9.5	48.7 ± 9.3
Sex, Men	23(85.8)	22(78.6)
Smokers	5(18.5)	4(12.3)
Ex-smokers	3(11.1)	4(12.3)
Previous coronary artery disease	9(33.3)	9(32.1)
Previous angina pectoris	6(22.2)	3(10.7)
Hypertension	12(44.4)	14(50.0)
Diabetes mellitus	8(28.6)	10(35.7)
Thrombolysis	2(7.4)	2(6.2)
Congestive heart failure at presentation	13(47.1)	12(36.9)
Site of acute myocardial infarction (AMI)		
Anterior	20(74.1)	24(85.7)
Inferior	16(59.2)	13(44.4)
Peak creatinine Kinase(IU/L)	1050 ± 292	915 ± 300

Values are mean ± standard deviation and numbers (%), AMI = acute myocardial infarction.

**Table 3:** Heart rate and blood pressure response.

Clinical Data	Intervention (*n* = 27)	Control Group (*n* = 28)
Before discharge		
Heart rate	74.1 ± 12.6 **	94.2 ± 15.5
Systolic blood pressure	110.5 ± 10.6 **	127.6 ± 16.2
Diastolic blood pressure	72.6 ± 9.5 *	83.5 ± 10.5
After 6 month		
Heart rate	64.0 ± 9.6 **	84.2 ± 13.6
Systolic blood pressure	116.2 ± 14.4 *	127.1 ± 16.5
Diastolic blood pressure	79.2 ± 8.5 *	88.4 ± 8.6

* *p* < 0.05, ** *p* < 0.02, *p* value was obtained by comparison of data before and after treatment by analysis of variance. Values are mean ± standard deviation.

**Table 4:** Changes in left ventricular dysfunction on two dimensional echocardiography.

Echocardiographic Data	Intervention Group (*n* = 27)		Control Group (*n* = 28)	
	Baseline	After 6 Months	Baseline	After 6 Months
End diastolic volume (mL)	94.2 ± 1.9	90.5 ± 2.0 **	97.5 ± 1.9	104.4 ± 2.2
End systolic volume (mL)	52.5 ± 1.5	48.0 ± 1.2 *	55.7 ± 1.7	59.7 ± 1.8
Left ventricular wall	12.0 ± 2.0	10.0 ± 1.8	12.8 ± 2.2	13.3 ± 2.3
thickness (mm)				
Left ventricular mass (g)	236 ± 72	213 ± 61 **	230 ± 77	255 ± 86
Area of wall thickening at	9.4 ± 3.0	9.1 ± 2.8	10.1 ± 3.1	13.7 ± 4.2
the site of infarct (m^2^)				
Sphericity index	1.61 ± 0.28	1.63 ± 0.30	1.61 ± 0.32	1.41 ± 0.31

* *p* < 0.05, ** *p* < 0.01, *p* value was obtained by comparison of intervention group with control group. All continuous variables are expressed as mean ± standard deviation. Analysis of variance followed by post–hoc Tukey’s test was performed to investigate the effect of the coenzyme Q10 compared to the placebo.

**Table 5:** Changes in biochemical data after 6 months.

Laboratory Data	Intervention Group (*n* = 27)		Control Group (*n* = 28)	
	Baseline	After 6 Months	Baseline	After 6 Months
Vitamin E (mg/dL)	15.5 ± 2.5	30.5 ± 4.2 **	14.4 ± 2.4	21.5 ± 3.5
Vitamin C (mg/dL)	4.5 ± 1.21	32.1 ± 4.0 **	4.6 ± 1.2	12.6 ± 2.5
Beta carotene (mg/dL)	0.16 ± 0.03	0.45 ± 0.07 *	0.15 ± 0.04	0.27 ± 0.05
TBARS (nmol/ml)	2.5 ± 0.51	1.2 ± 0.32 *	2.6 ± 0.42	2.4 ± 0.00
Malondialdehyde (nmol)	3.4 ± 0.32	2.0 ± 0.41 *	3.5 ± 0.35	3.0 ± 0.31
Diene conjugates (OD units)	33.5 ± 4.6	28.4 ± 4.0	33.6 ± 4.8	31.5 ± 4.5
Angiotensin converting enzyme (IU)	108.2 ± 13.5	70.6 ± 8.5 **	104.5 ± 11.4	92.6 ± 9.7

* *p* < 0.02 ** *p* < 0.01, *p* value was obtained by comparison of post-treatment values in the two groups and between baseline and post-treatment values in each group by analysis of variance followed by post hoc Tukeys test.
